# Risk, Trust, and Flawed Assumptions: Vaccine Hesitancy During the COVID-19 Pandemic

**DOI:** 10.3389/fpubh.2021.700213

**Published:** 2021-07-01

**Authors:** Omid V. Ebrahimi, Miriam S. Johnson, Sara Ebling, Ole Myklebust Amundsen, Øyvind Halsøy, Asle Hoffart, Nora Skjerdingstad, Sverre Urnes Johnson

**Affiliations:** ^1^Department of Psychology, University of Oslo, Oslo, Norway; ^2^Modum Bad Psychiatric Hospital, Vikersund, Norway; ^3^Department of Behavioural Science, Oslo Metropolitan University, Oslo, Norway; ^4^Department of Clinical Psychology, University of Bergen, Bergen, Norway

**Keywords:** vaccine hesitancy, general adult population, COVID-19 pandemic, information platforms, erroneous beliefs, psychological predictors, risk perception, governmental trust

## Abstract

**Background:** The pace at which the present pandemic and future public health crises involving viral infections are eradicated heavily depends on the availability and routine implementation of vaccines. This process is further affected by a willingness to vaccinate, embedded in the phenomenon of vaccine hesitancy. The World Health Organization has listed vaccine hesitancy among the greatest threats to global health, calling for research to identify the factors associated with this phenomenon.

**Methods:** The present cross-sectional study seeks to investigate the psychological, contextual, and sociodemographic factors associated with vaccination hesitancy in a large sample of the adult population. 4,571 Norwegian adults were recruited through an online survey between January 23 to February 2, 2021. Subgroup analyzes and multiple logistic regression was utilized to identify the covariates of vaccine hesitancy.

**Results:** Several subgroups hesitant toward vaccination were identified, including males, rural residents, and parents with children below 18 years of age. No differences were found between natives and non-natives, across education or age groups. Individuals preferring unmonitored media platforms (e.g., information from peers, social media, online forums, and blogs) more frequently reported hesitance toward vaccination than those relying on information obtainment from source-verified platforms. Perceived risk of vaccination, belief in the superiority of natural immunity, fear concerning significant others being infected by the virus, and trust in health officials' dissemination of vaccine-related information were identified as key variables related to vaccine hesitancy.

**Conclusion:** Given the heterogeneous range of variables associated with vaccine hesitancy, additional strategies to eradicate vaccination fears are called for aside from campaigns targeting the spread of false information. Responding to affective reactions in addition to involving other community leaders besides government and health officials present promising approaches that may aid in combating vaccination hesitation.

## 1. Introduction

The development of vaccines is considered among the greatest scientific achievements in promoting global health (1–3), with vaccination programs decreasing the burden of infectious diseases, loss of life, and contributing to strengthening the health system as a whole (4–6). However, reaping such benefits depends upon a willingness to vaccinate, as encapsulated in the phenomenon of vaccine hesitation, referring to a delay in acceptance or refusal of vaccines despite availability of vaccination services (7). Vaccine hesitancy is according to the WHO (7) among the top 10 threats to global health, with the necessity of identifying the psychological and sociodemographic factors interconnected with such hesitancy deemed as urgent. Additionally, the public's dependability on an efficacious vaccine has been alarmingly demonstrated during the present pandemic, with the pace of the pandemic's course directly interwoven with the availability and routine implementation of vaccines. Meanwhile, the procedural implementation of mass vaccination programs is heavily dependent on low vaccine hesitancy, with the phenomenon becoming a growing concern in relation to emerging infectious diseases and further being considered as a major threat toward the successful roll-out of vaccines during the present and forthcoming pandemics (8).

Parallel with the rapid and ongoing developments of several vaccines against COVID-19 globally, recent studies on hesitancy toward the COVID-19 vaccines have provided preliminary insight concerning some of the covariates characterizing vaccine hesitant individuals. The literature has hitherto indicated that those who perceive COVID-19 as a severe disease have a higher vaccination intention (9). Studies have also indicated that the novelty and rapid development of vaccines may have impacted the perceived risk of vaccination, with highlighted fearful cognitions concerning the pace and novel technologies utilized (i.e., mRNA vaccines) contrasting with conventional vaccine development approaches (10). Among promotive factors, trust in health officials has been highlighted as a possible factor which may decrease individuals' perception of vaccination risks (11). Despite these preliminary advances, several factors of importance remain uninvestigated.

The hesitancy to vaccinate is theorized to be connected to a broad array of cognitive, social, and contextual factors. Subjective risk perception of vaccine acceptance (12) may be affected by psychological dispositions, fear of side effects, health-related fears, beliefs and cognitions (13), and confirmatory biases involving pursuance of information congruous with one's established beliefs about vaccines (14, 15). Questioning the legitimacy of science (16), trust in government and health care officials, increased accessibility of dramatic individual experience across social media platforms, and major public health and vaccine scares in the media include factors of relevance that may covary with vaccination hesitation (13). Furthermore, the relationship between general adherence to pandemic mitigation protocols and vaccination intention warrants investigation given the association between other preventive behaviors, including social distancing and hygienic behaviors (17, 18). Another plausible factor of importance concerns the source of obtained information about vaccines and the pandemic. With the ubiquitous presence of news on social media, there are increased concerns about the reliability and accuracy of information (19), with a previous study suggesting social media platforms to be more biased toward false, misleading, and conspiratorial information about COVID-19 vaccines (20). The present study thus seeks to empirically investigate the association between vaccine hesitancy and these theorized factors, adapting a multifactorial approach in order to identify the relative importance of included predictors while controlling for all other variables in the analyzes. Consequently, the strongest factors associated with vaccine hesitancy are identified, presenting important implications for public health during the present and forthcoming pandemics.

## 2. Materials and Methods

This report is carried out in conformity with the guidelines of the Strengthening the Reporting of Observational Studies in Epidemiology statement [STROBE; (21)]. Ethical approval of the study was granted by The Regional Committee for Medical and Health Research Ethics (reference: 125510).

### 2.1. Participants and Procedure

The present article is part of The Norwegian COVID-19, Mental Health and Adherence Project, consisting of data from the fourth wave of data collection. At the first wave of data collection (i.e., between March 31 and April 7, 2020), 10,061 participants were recruited through an online survey disseminated via national, regional, and local information platforms (i.e., television, radio, and newspapers), in addition to dissemination to a random selection of Norwegian adults through a Facebook Business algorithm. This procedure is elaborated in detail elsewhere (22). All respondents were re-contacted for participation for the fourth wave of data collection between January 23 and February 2, 2021, of which 4,571 were eligible for the present study. The criterion variable of the present study (i.e., vaccine hesitancy) was measured during the fourth wave of data collection, thus yielding a cross-sectional study consisting of responses at the fourth wave of the project. Eligible participants included all individuals of 18 years and above (i.e., adults), who were living in Norway and thus experiencing identical social distancing protocols. The interval between January 23 and February 2, 2021 characterized a period where strict social distancing protocols had been re-implemented to a maximum. Among the implemented protocols were restrictions of leaving one's home unless necessary, home isolation if infected, quarantine after exposure to possible infection, restrictions on traveling, and prohibitions of social gatherings and events.

### 2.2. Study Design

The present cross-sectional study was designed to control for the impact of viral mitigation protocols (i.e., physical distancing protocols), in addition to expectation effects with respect to these strategies. Consequently, a stopping rule for data collection was construed to end collection immediately if viral mitigation protocols were modified or if new information about forthcoming modifications were provided. The data collection period was planned to last for 14 days, but was interrupted after 11 days with respect to the stopping rule as novel information about modification of mitigation protocols was provided (23), thus keeping expectation effects constant.

### 2.3. Measurement

#### 2.3.1. Demographic Information

The participants reported their age, sex, education, ethnical background, employment sector, parental status, and residency in urban vs. rural areas.

#### 2.3.2. Measurement of Vaccine Hesitancy and Vaccine-Relevant Beliefs

Vaccine hesitancy was measured by asking participants whether they planned to vaccinate themselves against COVID-19 when offered a vaccine on a binary scale (Yes vs. No), with individuals responding ‘No' being coded as hesitant toward vaccination.

Perceived risk of vaccination was measured by asking participants how risky they judged vaccinating themselves against COVID-19 on a continuous scale ranging from 0 to 100, with the left anchor labeled “No risk at all” and the right anchor labeled “Maximum risk.”

Participants' beliefs in the superiority of natural immunity as compared to vaccination was measured by asking them to rate the statement “Overall, I believe it is less risky or dangerous for me to obtain natural immunity as compared to vaccinating myself against the coronavirus”, rated on a five-point Likert scale (1–5; 1 = Completely Disagree, 5 = Completely Agree). Participants further reported their degree of trust in the vaccination information disseminated by the government and health-care officials on a five-point Likert scale (1–5; 1 = Not at all, 5 = Completely).

#### 2.3.3. Other Psychological and Contextual Variables

The Obsession with COVID-19 Scale [OCS; (24)] was used to measure persistent and obsessive thinking about COVID-19, consisting of a five-point Likert scale (0: Not at all to 4: Nearly every day over the last 2 weeks). The internal consistency of this scale was good in this sample, with a Cronbach's alpha of 0.78.

Fear of significant others being infected was measured with the item "I fear that someone close to me may contract the coronavirus," rated on a four-point Likert scale (0–3; 0 = Not at all, 4 = Nearly every day).

Participants were further asked whether they were subject to strict social distancing protocols, including visitation regulation, quarantine, and isolation, providing the duration of which they were subject to these protocols in the unit of weeks. The time-scale of the length of exposure to strict distancing protocols was transformed from weeks to months.

Media preference source was measured by asking participants to disclose their preferred media platform in obtaining information about the pandemic, with source-verified platforms consisting of source-checked and recognized national, regional, and local television, newspapers, and radio channels, and unmonitored sources consisting of social media platforms (e.g., Instagram, Snapchat, Tiktok), online forums and blogs, and preferred information obtainment from friends, family and peers.

Adherence to pandemic mitigation protocols was measured by asking participants how well they were able to adhere to the hygienic behavior-recommendation (e.g., covering mouth and nose with a tissue or elbows when coughing and sneezing, and avoidance of touching the eye, nose and mouth area) and the social distancing protocols (e.g., maintaining one meter distance to individuals not in one's household) implemented in Norway at the time of data collection, measured on a continuous scale ranging from 0 to 100, with the left anchor labeled “Not at all (0% of the time)” and the right anchor labeled “Fully (100% of the time).” All fully continuous measures were re-scaled from a 0–100 to a 0–10 scale for ease of interpretation.

### 2.4. Statistical Analyzes

All statistical analyzes were performed using R [Version 4.0.2; (25)]. Descriptive analyzes concerning subgroups were reported applying percentages and proportions, using chi-square statistics to test differences between subgroups. To inspect the predictors of vaccination hesitancy, a two-step hierarchical logistic regression was conducted with vaccine hesitancy as the criterion variable. The first step of the model involved the demographic background variables (i.e., sex, age, and education) to control for the potential confounding influence of these, in addition to identifying the demographic risk factors associated with the criterion variable. The second step of the model included the mentioned variables (see Measurement section) concerning vaccine-relevant beliefs, contextual, and psychological variables. Improvements in model fit was evaluated using the Akaike Information Criterion (AIC). Odds ratios along with their 95% confidence intervals are provided. To further estimate model fit and explanatory power, the confusion matrix was inspected, with the diagonal revealing the correctly identified cases of vaccination hesitancy and vaccination intention given the specified model as compared to the observed data.

## 3. Results

### 3.1. Sample Characteristics

The sample consisted of 4,571 participants, with the age ranging from 18 to 86 with a mean of 36.66 years (*SD* = 13.72). The demographic details of the participants is presented in Table 1. Approximately 78% of the sample were females, with all analyzed subgroups richly represented in the study (e.g., *N* = 999 males), and sensitivity analyzes on the same group of participants [see, (22)] revealing the sample as accurate and representative for the general adult population following a) analysis solely on the randomly selected proportion of participants, in addition to b) analysis on an adjusted, post-stratified and weighted sample, both of which replicated and demonstrated indifferent results as the main sample. The sample was further geographically representative of Norway, with the ratio of individuals from each region closely approximating the population distribution.

**Table 1:** Demographic characteristics.

All participants	
*N*	4,571 (100.00%)
Sex	
Female	3,560 (77.88%)
Male	999 (21.86%)
Education	
Completed Junior High School	227 (4.97%)
Completed High School	807 (17.65%)
Currently studying	911 (19.93%)
Completed University Degree	2,626 (57.45%)
Age group, years	
18–30	1971 (43.12%)
31–44	1369 (29.95%)
45–64	1037 (22.69%)
65+	194 (4.24%)

### 3.2. Vaccine Hesitancy Among Subgroups

Overall, 478 (10.46%) participants reported being hesitant toward vaccination. Table 2 presents the proportions and percentages of specific subgroups' vaccine hesitancy. Individuals with a preference for unmonitored media platforms as compared to those preferring source-verified media platforms had a near 2-fold (i.e., 1.64) odds of being hesitant toward vaccination. Rural residents had a near 2-fold (i.e., 1.71) odds of being hesitant toward vaccination compared to urban residents. Parents with children aged below 18 had 1.46 times higher odds of reporting vaccine hesitancy. Health-sector employees were less hesitant toward vaccination by a factor of 0.78. No difference was found between immigrants and natives concerning vaccine hesitancy.

**Table 2:** Proportions and percentages of different subgroups expressing hesitation toward vaccination.

	***N***	**Hesitancy^a^**	**Chi-square test**
**All participants**	4,571	10.46%	
**Health-care sector employee**			*χ^2^*(1, *N* = 4,571) = 6.10, *p* = 0.014
Yes	1,301	8.67%	
No	3,270	11.16%	
**Natives and immigrants**			*χ^2^*(1, *N* = 4,571) = 0.74, *p* = 0.385
Immigrants	270	8.89%	
Natives	4301	10.56%	
**Rural and urban residency**			*χ^2^*(1, *N* = 3,431) = 13.93, *p* < 0.001
Urban resident	2943	9.11%	
Rural resident	488	15.55%	
**Media preference**			*χ^2^*(1, *N* = 3,354) = 13.58, *p* < 0.001
Source-verified media platforms	2,937	9.13%	
Unmonitored media platforms	417	14.87%	
**Has children below 18 years**			*χ^2^*(1, *N* = 4,571) = 18.63, *p* < 0.001
Yes	1,484	13.28%	
No	3,087	9.10%	

a *Percentage of subgroup hesitant toward vaccination*.

### 3.3. Psychosocial Predictors of Vaccine Hesitancy

The hierarchical logistic regression model investigating predictors of vaccine hesitancy may be found in Table 3. The confusion matrix revealed excellent model fit and performance, with the specified model correctly predicting 92.30% of the cases of vaccination hesitancy and vaccination intention. The model substantially improved from Step 1 to Step 2 with the AIC equal to 3,056 at Step 1 and 1,722 at Step 2. Among the demographic variables, only sex was significantly associated with vaccine hesitancy, with the odds of males being hesitant toward vaccination larger by a factor of 1.29 as compared to females. Age and education were unrelated to vaccination hesitancy.

**Table 3:** A hierarchical logistic regression model revealing the predictors of vaccine hesitancy.

**Step**	**Predictor**	**Logit**	***SE***	**OR (95% CI)**	***p***
1	**Demographic control variables**				
	Sex^a^	0.252	0.114	1.287 (1.026, 1.605)	0.027
	Age	0.002	0.004	1.002 (0.995, 1.009)	0.608
	Education	–0.060	0.052	0.942 (0.852, 1.044)	0.248
2	**Psychological and contextual variables**				
	Perceived risk of vaccination	0.287	0.026	1.332 (1.265, 1.404)	<0.001
	Belief in superiority of	0.980	0.065	2.663 (2.350, 3.028)	<0.001
	natural immunity				
	Trust in disseminated information	–0.772	0.067	0.462 (0.405, 0.526)	<0.001
	about vaccination from				
	health officials				
	Obsession with COVID-19	–0.057	0.029	0.945 (0.892, 1.000)	0.054
	Fear of significant	–0.344	0.095	0.709 (0.587, 0.853)	<0.001
	others being infected				
	by the virus				
	Adherence to pandemic	–0.105	0.042	0.900 (0.829, 0.977)	0.011
	strict distancing protocols				
	Length of exposure to	0.042	0.020	1.043 (1.002, 1.085)	0.041
	strict distancing protocols				

a *Male (1), Female (0)*.

Concerning the psychological and contextual variables, a one unit increase in perceived risk of vaccination increased the odds of vaccine hesitancy by 1.33. Stronger belief in superiority of natural immunity as compared to vaccination was associated with vaccine hesitancy, with a one-unit increase elevating the odds of vaccine hesitancy by a factor of 2.66. For a one unit increase in trust of disseminated information about vaccination from health officials and governments, the odds of being hesitant toward vaccination was smaller by a factor of 0.46. For a one unit increase in fearing that significant others may be infected by coronavirus, the odds of vaccine hesitancy was smaller by a factor of 0.71. Per months increase in having predominantly socially distanced oneself (e.g., as a result of quarantine), the odds of being hesitant toward vaccination increased by 1.04. For a one unit increase in adherence to pandemic mitigation protocols, the odds of vaccination hesitation was smaller by a factor of 0.90. Obsession with COVID-19 was unrelated to vaccine hesitancy.

## 4. Discussion

The pace of which the pandemic crisis is eradicated heavily depends on vaccination and the successful combating of vaccination hesitancy. Furthermore, incidences of public health scares related to vaccines as exemplified by the AstraZeneca public health scare (26) is likely to increase the observed rates of vaccine hesitancy. Thus, empirical findings concerning the predictors of vaccination hesitation, including the subgroups at greater risk for such hesitancy is of imperative value in the present and forthcoming pandemics.

The presented results indicate that around 11% of the sample displayed vaccine hesitancy, meaning that the majority of the sample (89%) intend to vaccinate when given the opportunity. The results portray somewhat less skepticism toward vaccination in the Norwegian population compared to other countries across Europe. Vaccine hesitancy rates in European countries in the first half of 2020 (i.e., February until the end of May) ranged from 17 to 38%, and in the second half of 2020 (i.e., June until December) ranged from 25 to 46% (8, 27). A plausible explanation for the lower prevalence of vaccination hesitancy in Norway includes the high level of trust in health and governmental officials in the Norwegian population and the Nordic countries in general (28, 29). Indeed, this was reflected in the aforementioned studies on vaccine hesitancy, identifying less frequent vaccine hesitancy in the Nordic countries compared to other nations.

Comparisons across subgroups led to the identification of the following groups more frequently reporting vaccination hesitancy: people not working in the health sector, those living in rural vs. urban districts, people preferring information obtained from non-monitored information sources rather than source-verified media platforms (e.g., national newspapers and television channels), and parents of children below 18 years of age. People in the health sector are likely more able to realistically evaluate the benefits and risks of vaccines and less prone toward the type of erroneous conclusions (e.g., belief in greater benefits of natural immunity) and overestimation of risks which were here found to be related to vaccine hesitation. Moreover, parents tend to feel more responsible for their children than for themselves and their perception of vaccine risk accordingly seems to be increased, echoing previous findings (30, 31). The finding that rural residents were more likely to be hesitant toward vaccination is further consistent with findings showing more vaccine resistance among people residing in the suburbs than in cities (32). Importantly, although highlighted as a concern in the literature (33, 34), the present study found no empirical support for the notion that immigrants display more skepticism toward vaccination compared to natives. This has been a theorized concern assuming less access to public information as a relevant factor (33, 34). Thus, the Norwegian governments implemented campaigns translating vaccination and pandemic-related information in the native languages of minority groups seems to have been an appropriate precautionary strategy in preventing the development of such forecasted problems.

Moreover, the importance of combating false information from unmonitored media sources is underscored in the present study, further accentuated through findings illustrating that exposure to as little as 5–10 min of negative and inaccurate information about vaccines increases the risk perception associated with vaccination (35). The present study extends the literature by empirically revealing that information obtainment from unmonitored platforms further is associated with vaccination hesitation, beyond and above the influence of such platforms on risk perception. Additionally, this association is found between other important types of information platforms other than social media. Thus, the present study highlights the importance of extending the efforts to prevent the spread of misinformation beyond social media platforms. Social media platforms will however continue to be an important battleground to hinder misinformation. Given lower levels of editorial oversight, social media use has previously been found to be related to overestimation of vaccine risks, thus explaining the greater likelihood of hesitancy among social media users (20, 36), revealing in conjunction with the present findings that it will be important to sustain the misinformation-prevention efforts directed at these ubiquitous platforms. The present study further found an association between a prevalent contextual factor, namely adherence to general pandemic protocols (e.g., hygiene-behavior adherence) and vaccination hesitancy, highlighting that those who adhere to pandemic protocols more likely to be associated with intentions to vaccinate, identifying non-adherent individuals as an in-risk group. It is however interesting to note that length of exposure to strict distancing protocols (e.g., isolation and quarantine) was associated with vaccination hesitation. A possible explanation of this finding may include that some of the individuals experiencing such strict protocols of prolonged length are those in isolation as a result of having contracted the disease, thus possibly developing beliefs of being immune to the disease. Given the possibility of multiple infections by the virus, efforts to clarify such misconceptions are imperative. In sum, the presented results point toward several subgroups that could benefit from tailored campaigns aimed at reducing vaccine hesitancy.

In examining the psychological predictors of vaccine hesitancy, central demographic characteristics were controlled for. Being male was slightly associated with hesitancy toward vaccination. This finding is diverges from a recent review exploring vaccine hesitancy and intention during the ongoing pandemic [see (37)], with more frequent hesitancy found among females compared to males. A possible explanation of this inconsistency could reflect the fact that Norwegian females to a greater extent than males seek out health services [Norwegian health service use (38)]. This finding could also be mirrored by the fact a larger proportion of females are employed in the Norwegian health sector, with health-care workers found to display less prevalent hesitance toward vaccination in a previous (39) and the present study.

The results further highlight the importance of considering the cognitive and emotional risk-benefit evaluation processes ongoing in individuals, with those perceiving that the risk of vaccination greater than the risk of being infected by the disease more likely to report hesitancy toward vaccination. Consistent with previous findings, increased risk perception was associated with vaccination hesitancy (9, 40). Moreover, the present study found specific belief sets to be related to vaccine hesitancy, with stronger belief that obtaining natural immunity is less dangerous than vaccinating being associated with vaccination hesitation. Given the stressed necessity of vaccination implementation procedures in eradicating the coronavirus (41), accurate information regarding the actual effect of natural immunity vs. vaccination and the associated risk factors should be disseminated to the general population. Achieving natural immunity through infection rather than vaccines puts individuals at greater risk when considering the reports of long-term complications after infection with COVID-19, and the large proportion of COVID-related deaths that follows a herd immunity strategy (42, 43). Thus, such maladaptive beliefs must be combated to impede the health-related risks for those withholding them in addition to society at large. In light of recent public health scares and the rapid pace at which information spreads (e.g., digitally), fearful and inaccurate vaccine beliefs are likely to increase and flourish across social media and other digital platforms (44). Thus, health officials must identify outlets and information channels where groups with such beliefs and more prevalent degrees of vaccine hesitancy are found and further given the possibility of dialogue, which may minimize the reactivity associated with distrusting the messenger and thus have the possibility to foster the grounds upon which inaccurate perceptions of vaccination risks may be corrected. Acknowledging fears, anger and other negative emotions while emphasizing the safety of COVID-19 vaccines may provide aid in reducing vaccine hesitancy. Indeed, several scholars have highlighted the limitation of communication strategies solely relying on fact checking, emphasizing the need for effective communication to address emotional responses and cognitive biases that may arise during pandemics [e.g., (45)]. Additional aid toward this aim may be achieved by appealing to altruism, framing vaccination as a step toward a meaningful goal for the society as a whole, creating a sense of communion in the battle against the virus (46).

The present study demonstrates that trust in disseminated information about vaccination from the government and health officials was associated with less frequent vaccination hesitancy. Clever strategies are required to reach individuals who distrust information from their government and health officials with accurate information concerning risks, which have the ability of modifying risk perceptions and beliefs about natural immunity. It is thus imperative for health officials to propagate such information in a manner which minimizes reactivity from the more distrusting and skeptical subgroup of the population. One possibly utile strategy may include information campaigns through platforms which information dodgers less typically avoid, such as the dissemination of information in-between entertainment programs (e.g., TV-series). This finding further highlights the importance of strengthening the government as a credible sender of information, something which is threatened by digital misinformation (47, 48). Importantly, other sources of information should be incorporated in the vaccine campaigns, such as religious or political leaders, healthcare workers, or other influential members of the community [European Centre for Disease Prevention and Control (49)], not solely leaning on the information dissemination by the government and health officials. Such a wide dissemination strategy may be a potent strategy in lowering vaccination hesitancy by increasing the probability of reaching out to skeptical and hesitant individuals through alternative sources which they trust.

Finally, this study reveals that individuals fearing that someone closely related to them could potentially be infected by the virus less frequently reported hesitance toward vaccination. Accordingly, the encouragement of the prosocial implications of vaccination and caring about the welfare of others may be a utile strategy of lowering hesitance, previously found to be positively associated with vaccination intent in the population [e.g., (50, 51)]. These results point toward lifting the focus from the individual costs of vaccination toward the negative consequences of non-vaccination for others (e.g., family members, elderly, people with underlying diseases) and society at large, which may further aid in reducing vaccine hesitancy.

### 4.1. Strengths and Limitations

The present has several limitations, including its cross-sectional design precluding causal conclusions, and that random selection was not conducted on the full sample due to the online dissemination strategy chosen. As this strategy plausibly is less accessible to elderly, we had to recruit elderly adults through broadcastings on national television, radios, and newspapers, thus leaving parts of the sample non-randomly selected. Notwithstanding, these efforts to provide the full adult population an equal opportunity to participate proved futile, with the resulting sample revealed as representative with no influence demonstrated in sensitivity analyzes by the few undersampled subgroups (e.g., less males and elderly) on the results (22). Given the lack of existing measures for some of the variables related to the research questions of this investigation, several variables were adapted and developed for the present study, thus being untested across other settings and serving as a limitation of this study. The study also includes several strengths, including the fit and performance of the model, large sample size, and simultaneous investigation of a multitude of relevant variables providing more robust estimates of factors while controlling for the impact of all relevant predictors. It is noteworthy that investigations involving sensitive criterion variables, including the present study on vaccine hesitancy, may be susceptible to the Hawthorne effect, which postulates that participants may alter their responses and attitudes toward a phenomenon when knowing they are being investigated. The potential presence of such an effect could thus have led to underestimations of vaccine hesitancy in the general population. However, the present study was designed to contain the set of sensitive questions concerning vaccination between larger sets of measures related to other phenomena (e.g., sleep difficulties), thus increasing the chance of suppressing such reactivity given the efforts undertaken to mask the research question.

### 4.2. Concluding Remarks

Vaccine hesitancy is interwoven with an array of psychological, demographic, and contextual factors, including political and systemic factors. The phenomenon is tied to impeding pandemic eradication, in addition to increasing the risk of vaccine-preventable disease and death toll during pandemics. Thus, the empirical underpinnings of vaccine hesitancy are not only of importance during the present, but also forthcoming pandemics.

The present study investigated a multitude of factors associated with vaccine hesitancy, laying the grounds for possible strategies that may be of benefit in reducing hesitation and worth pursuing in upcoming studies for further validation. On the individual level, the present findings suggest that emphasis should be placed on the perceived risk of vaccination, and further highlight the importance of combating the inaccurate assumption of superiority of natural immunity. Interventions on the community level impact larger numbers of individuals and should be prioritized. At this level, proposed ways to counter vaccine hesitancy includes transparency in policy-making decisions regarding the vaccination program and clear provision of information about the rigorous process that underlie the approval of new vaccines. Given the highly heterogenous covariates associated with vaccination hesitancy, additional cues besides provision of rational and fact-based information are called for. Finally, to compensate for the detrimental association between vaccination hesitancy and low trust in governments and health officials, the involvement and aid of other community leaders are called for and of importance.

## Data Availability Statement

Our received ethical approval precludes us from submitting the data to a public repository. In line with the ethics approval, the data are to be kept at a secure server only accessible by the authors at the University of Oslo. Access to the data can be granted from the principal investigators Omid V. Ebrahimi and Sverre Urnes Johnson following ethical approval of a suggested project plan for the use of data from NSD and REK. Requests to access the datasets should be directed to omideb@uio.no.

## Ethics Statement

The studies involving human participants were reviewed and approved by The Regional Committee for Medical and Health Research Ethics (reference: 125510). The patients/participants provided their written informed consent to participate in this study.

## Author Contributions

OVE designed the study, conducted the data collection, and performed the data analyzes under the supervision of AH and SJ. The literature review was conducted by OVE, SE, NS, MJ, OMA, and ØH. All authors contributed to the discussion of the results in addition to writing and revision the manuscript under the supervision of OVE and SJ. The final revision and preparatory work of the manuscript was done by OVE. OMA and OVE formatted the manuscript for submission.

## Conflict of Interest

The authors declare that the research was conducted in the absence of any commercial or financial relationships that could be construed as a potential conflict of interest.
